# Supplementary material for: Fatigue Is a Major Symptom at COVID-19 Hospitalization Follow-Up
Source: J Clin Med. 2022 Apr 25;11(9):2411. doi: 10.3390/jcm11092411 (PMC9106038; doi:10.3390/jcm11092411)
Supplement: Supplementary file 1 [file jcm-11-02411-s001.zip › jcm-1668151-supplementary.pdf]

Supplementary

Table S1. Additional basic demographic characteristics during hospitalization

	Overall. *n*=218	Patients not requiring supplemental oxygen (group 1). (*n*=57)	Patients requiring supplemental oxygen (group 2) (*n*=120)	Patients admitted to ICU (group 3) (*n*= 41)
Comorbidities				
Ischemic heart disease	16 (7%)	6 (11%)	5 (5%)	5 (12%)
Cerebrovascular disease	9 (4%)	1 (2%)	7 (6%)	1 (2%)
Kidney disease	5 (2%)	0 (0%)	4 (3%)	1 (2%)
Immunodeficiency	2 (1%)	2 (4%)	0 (0%)	0 (0%)
Liver disease	1 (0%)	0 (0%)	1 (1%)	0 (0%)
Organ transplant	1 (0%)	0 (0%)	1 (1%)	0 (0%)
Other	102 (47%)	24 (42%)	63 (53%)	15 (37%)
Smoking status				
Never smoker	106 (54%)	31 (57%)	60 (57%)	15 (39%)
Former smoker	87 (44%)	21 (39%)	44 (42%)	22 (58%)
Current smoker	5 (3%)	2 (4%)	2 (2%)	1 (3%)
Treatment during hospitalization				
Intravenous fluids	124 (58%)	27 (47%)	66 (55%)	31 (82%)
Antibiotics	104 (48%)	17 (30%)	49 (41%)	38 (95%)
Fungal treatment	27 (13%)	2 (4%)	5 (4%)	20 (53%)
Dialysis	6 (3%)	0 (0%)	2 (2%)	4 (10%)

Data are n (%).

Table S2. Additional characteristics for patients hospitalized for PCR-proven Covid-19 at follow-up

	Overall. n=218	Patients not requiring supplemental oxygen (group 1). (*n*=57)	Patients requiring supplemental oxygen (group 2) (*n*=120)	Patients admitted to ICU (group 3) (*n*= 41)
Symptoms at follow-up				
Expectoration	34 (16%)	8 (14%)	15 (13%)	11 (27%)
Runny nose	27 (12%)	4 (7%)	14 (12%)	9 (22%)
Loss of appetite	20 (9%)	8 (14%)	7 (6%)	5 (12%)
Stomach pain	19 (9%)	5 (9%)	9 (8%)	5 (12%)
Diarrhea	19 (9%)	4 (7%)	8 (7%)	7 (17%)
Nausea	18 (8%)	6 (11%)	8 (7%)	4 (10%)
Throat pain	14 (6%)	7 (12%)	4 (3%)	3 (7%)

Data are n (%).

Table S3. Additional demographics and outcomes at follow-up stratified for treatment with RaD

	Oxygen-dependent patients **not** receiving systemic corticosteroids and remdesivir (RaD÷) *n*=52	Oxygen-dependent patients receiving systemic corticosteroids and remdesivir (RaD+) *n*=86
Comorbidities		
Hypertension	16 (31%)	34 (39%)
Asthma	6 (12%)	19 (22%)
Diabetes	3 (6%)	21 (24%)
Malignancy	5 (10%)	7 (8%)
COPD	3 (6%)	8 (9%)
Symptoms at follow-up		
Fatigue	26 (50%)	52 (60%)
Dyspnea	22 (42%)	47 (55%)
Concentration impairment	13 (25%)	28 (33%)
Muscle pain	13 (25%)	28 (33%)
Coughing	13 (25%)	25 (29%)
Pulmonary function		
FEV1, L/min	2.92 (2.67, 3.16)	2.84 (2.65, 3.02)
FVC, L/min	3.85 (3.55, 4.15)	3.59 (3.38, 3.81)
TLC, %	96.21 (91.42, 100.99)	89.54 (84.37, 94.72)
RV, %	100.35 (93.54, 107.17)	94.86 (84.60, 105.11)
6MWT		
Percent predicted, men	81.53 (74.08, 88.99)	83.57 (78.42, 88.73)
Percent predicted, female	94.91 (85.06, 104.77)	98.79 (89.87, 107.73)
Desaturation below 92	7 (16%)	17 (24%)
Desaturation ≥4%-point	8 (18%)	25 (35%)

Data are n(%), mean (95% CI).
